# Amino terminal tyrosine phosphorylation of human MIXL1

**DOI:** 10.1186/1750-2187-1-6

**Published:** 2006-12-05

**Authors:** Wei Guo, Lalitha Nagarajan

**Affiliations:** 1 Department of Molecular Genetics, M.D. Anderson Cancer Center, University of Texas, Houston, Texas 77030, USA

## Abstract

Seven members of the Mix family of paired-type homeoproteins regulate mesoderm/endoderm differentiation in amphibians. In mammals, the *MIXL1 *(Mix. 1 homeobox [*Xenopus laevis*]-like gene 1) gene is the sole representative of this family. Unlike the amphibian Mix genes that encode an open reading frame of >300 amino acids, mammalian *MIXL1 *encodes a smaller protein (~230aa). However, mammalian MIXL1 contains a unique proline-rich domain (PRD) with a potential to interact with signal transducing Src homolgy 3 (SH3) domains. Notably, human MIXL1 also contains a unique tyrosine residue Tyr20 that is amino-terminal to the PRD. Here we report that mammalian MIXL1 protein is phosphorylated at Tyr20 and the phosphorylation is dramatically reduced in the absence of PRD. Our findings are consistent with Tyr20 phosphorylation of MIXL1 being a potential regulatory mechanism that governs its activity.

## Background

*Mix. 1*, a paired-like homeobox gene, was initially identified as an inducer of ventral mesoderm and/or endoderm in *Xenopus *[1,2]. Subsequently, several closely related genes, Mix 2–4, Bix 1–4, and Mixer, were isolated and found to regulate mesoderm and/or endoderm formation [3-6]. However, in chicken (CMIX), mice (MIXL1/Mml) and humans, the Mix-like homeobox (*MIXL1*) genes appear to be single copies [7-12]. Additionally, mammalian *MIXL1 *encodes a smaller protein of ~230 amino acids, in contrast to the ~340 amino acid proteins encoded by the *Xenopus *genes. Nonetheless, almost all of the Mix family members are modular with a highly conserved paired-type homeodomain and a conserved carboxy-terminal acidic domain (CAD). A distinguishing feature of CMIX, Mml/MIXL1 and human MIXL1 is the presence of proline-rich domains (PRD). Both mouse and human MIXL1 contain an amino-terminal PRD between residues 31–60; in chicken however, the PRD appears to be carboxyl to the homeodomain raising the possibility that the function of this domain may be modular.

The *Xenopus *Mix/Bix genes are expressed in ventral mesoderm and/or endoderm [1,3-6]. Similarly, the expression of mouse *Mml/MIXL1 *or chicken *CMIX *initially occurs in visceral endoderm and, becomes restricted to primitive streak and nascent mesoderm at gastrulation; in mice, this includes the hemangioblast, a precursor of hematopoietic and vascular stem cells [9-14]. The expression of human *MIXL1 *is restricted to progenitors and secondary lymph tissues in adults [8]. The temporal and spatial expression pattern of Mix-like genes suggests that these genes are tightly regulated during embryonic development and hematopoietic differentiation. The MIX family appears to be regulated by at least three signaling pathways: TGFβ/Activin/BMP, FGF, and p53 [2-4,6,15-19].

A role for the Mix gene family in development is suggested by both gain-and loss-of-function experiments. *Xenopus Mix.1 *gene is implicated in the process of patterning ventral mesoderm to hematopoietic fate induced by BMP-4 [15] and in endoderm development by synergizing with other regulatory molecules such as *Siamois *[2]. Similar to Mix.1, ectopic expression of human *MIXL1 *induced embryonic hematopoiesis in *Xenopus *animal caps[8]. Homozygous disruption of mouse *Mml*/*MIXL1 *resulted in a marked thickening of the primitive streak, severe defects in paraxial mesoderm, and absence of heart tube and gut [20]. *In vitro *ES differentiation assays further demonstrated that murine *Mml/MIXL1 *to be BMP4 responsive and required for efficient hematopoiesis [14].

In contrast to the developmental studies on this gene family, nothing is known about biochemical pathways regulating mammalian MIXL1. Absence of multiple family members coupled with the gain of PRD in mammals, raises a number of mechanistic possibilities for similar cell fate or differentiation pathways regulated in amphibians and mammals. One of these may be tissue- or developmental stage-specific phosphorylation of MIXL1 that may mimic the diverse regulatory functions by multiple members in *Xenopus*. In this report, we show that mammalian MIXL1 protein is readily phosphorylated at the amino terminal tyr20. Tyr20 phosphorylation of MIXL1, a potential regulatory mechanism governing its activity is dramatically reduced in the absence of PRD.

## Materials and methods

### Plasmid construction and mutagenesis

The full-length human MIXL1 ORF, ΔCAD mutants with truncation of carboxy-terminal acidic domain and ΔN25 mutant with truncation of both amino-terminal 25 bp region and carboxy-terminal acidic domain were amplified by PCR and cloned into expression vectors CMV5 (a kind gift from David W. Russell, UTSW at Dallas, TX) and CMV2-flag (Sigma, St. Louis, MO) to generate CMV5-MIXL1, CMV2-Flag-MIXL1, CMV5-ΔCAD, CMV2-flag-ΔCAD and CMV2-flag-ΔN25. The amino terminal of CMV2-flag-ΔCAD was replaced with the 1–93 bp amino-terminal portion of MIXL1 to generate the construct CMV2-flag-ΔPC lacking both PRD and CAD domains. Y110F mutation was introduced by PCR with a primer containing an A-to-T (Tyr-to-Phe) point mutation. The MIXL1 carboxy-terminal portion (301–699) of the construct CMV2-flag – ΔCAD or CMV2-flag-ΔN25 was replaced with the amplified MIXL1 fragments carrying the A-to-T point mutation to generate the construct CMV2-flag-Y110F or CMV2-flag-Δ2Y. Accuracy of the generated constructs was confirmed by double stranded sequencing.

### Cell culture and transfection

HEK 293T cells were grown in modified Eagle's medium (MEM, Invitrogen) supplemented with 10% FBS, 0.1 mM non-essential amino acids and 1 mM sodium pyruvate (Invitrogen) at 37°C in 10% CO2. For transfection, cells were plated at a density of 2 × 10^5 ^cells per well in a 6-well plate 2 days prior to transfection. The transfections were performed using LipofectAmine (Invitrogen) according to the manufacturer's protocols. Total amount of transfected DNA was adjusted to 1 μg per well by using appropriate parental vectors. Cells were harvested for nuclear extraction 48 hours after transfection. For immunoprecipitation experiments, the transfections were scaled up to 100 mm plates.

### Immunoprecipitation and immunobloting

Nuclear extracts were prepared from transfected 293T cells [8] and diluted to approximately 1.0 μg protein/μL lysate. Briefly, 500 μL of the fresh nuclear extracts were pre-cleared with 50 μL of protein-A-agarose bead slurry (50% v/v, Roche, Indianapolis, IN) at 4°C for 30 minutes in an orbital shaker. After centrifugation at 14,000 × g at 4°C for 10 minutes, supernatant was mixed with 5 μg of the murine monoclonal antibody 4G10 (Upstate) or the isotypic control (murine monoclonal antibody against the V5 epitope-Invitrogen) at 4°C overnight on the orbital shaker. The immune-complexes were captured by adding 50 μL of protein A agarose bead slurry (50% v/v) and gently rocking on the orbital shaker at 4°C for 2 hours. After pulse centrifugation at 14,000 rpm for 8 seconds, the immunecomplex-protein-A-agarose-bead pellets were collected and washed 5 times with 800 μL of ice-cold modified radioimmunoprecipitation (RIPA) buffer (50 mM Tris-HCl [pH 7.4]; 150 mM NaCl; 1% NP-40; 0.25% sodium deoxycholate; 1 mM EDTA; 1 mM PMSF; 2 μg/mL leupeptin; 2 μg/mL pepstatin A; 2 μg/mL aprotinin; 500 μg/mL benzamidine; 1 mM Na3VO4; 1 mM NaF) and once with 800 μL ice-cold 1× PBS. The pellets containing immune complexes were resuspended in 60 μL of 2× sample buffer (100 mM Tris-HCl [pH 6.8], 200 mM DTT, 4% SDS, 0.2% Bromophenol Blue, 20% Glycerol).

Immunoprecipitates or nuclear proteins were resolved (50 μg of protein per lane) on pre-cast 10% NuPAGE gels (Invitrogen). After electrophoresis, the proteins were transferred to Hybond P nylon membrane (Amersham Biosciences, Piscataway, NJ) at 30 V overnight. The protocol for immunobloting was essentially as detailed elsewhere [21]. Rabbit polyclonal antibody anti-MIXL1-N [8] was used at a dilution of 1:100 initially or 1:500 for immunoblotting studies. Mouse monoclonal antibody anti-flag M2 (Sigma) 1:300; mouse monoclonal antibody 4G10 (Upstate) 1:3000 or 1:4000.

### Alkaline phosphatase treatment

Nuclear extracts of HEK 293T cells transfected with CMV5-MIXL1 was prepared without the addition of phosphatase inhibitors sodium fluoride and sodium orthovanadate. Immediately after extraction, 15 μg of nuclear protein was incubated with 40 units of calf intestine alkaline phosphatase (CIAP, Roche) in a 20 μL reaction for 30 minutes at 30°C. As a control, the same reaction was performed in the presence of 100 mM sodium orthovanadate, which inhibits CIAP activity. In addition, a mock reaction was also performed with no CIAP. The reactions were terminated with NuPAGE sample buffer (Invitrogen). The changes in protein mobility were determined by probing the immunoblots with the anti-MIXL1-N antibody.

## Results and discussion

### MIXL1 is phosphorylated at multiple sites

**Figure 1:**
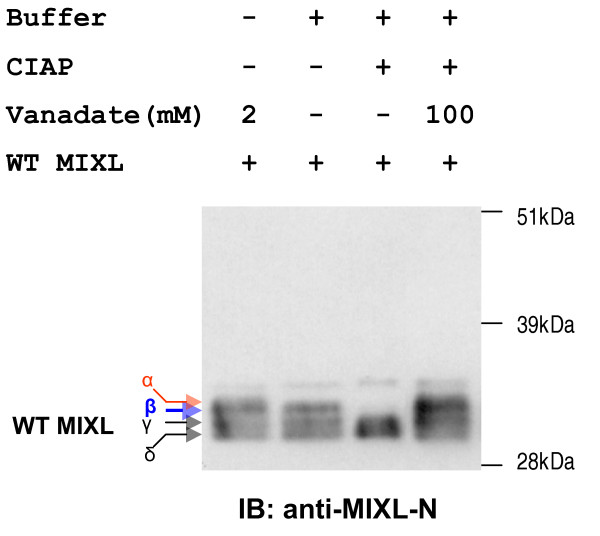
**Alkaline Phosphatase treatment of nuclear extracts alters the mobility of MIXL1 proteins on SDS-PAGE gel**. Expression plasmid pCMV5-MIXL1 was transiently transfected to HEK 293T cells and the transfectants were harvested 60 hours later. 15ug of fresh nuclear extracts were treated with 40u calf intestine alkaline phosphatase (CIAP) in the absence or presence of phosphatase inhibitor sodium orthovanadate at 30°C for 15 min. After CIAP treatment, nuclear extracts with orthovanadate and CIAP reactions were resolved on a NuPAGE gel and transferred to a PVDF membrane. Immunobloting was done with anti-MIXL1-N (1:350 dilution). Anti-MIXL1-N detected 4 species on lane 1 (nuclear extracts with orthovanadate and no CIAP treatment). The band α disappeared on mock reaction (lane 2). CIAP treatment resulted in disappearance of both band α and β (lane 3). In contrast, the addition of phosphatase inhibitor orthovanadate protected the all the 4 species (lane 4).

When the full-length *MIXL1 *expression driven under CMV promoter in the construct pCMV5-MIXL1 was tested by transient transfection into the HEK cell line 293T, we detected at least three species for MIXL1 proteins in nuclear extracts by immunobloting with the anti-MIXL1-N antibody. Similar experiments with other expression constructs containing full-length or truncated MIXL1 also detected at least two species for MIXL1 proteins. To test the possibility that the multiple bands were the MIXL1 proteins modified by phosphorylation, the nuclear extracts were treated with alkaline phosphatase (CIAP) to remove phosphate groups from phosphorylated proteins. MIXL1 proteins were detected by immunobloting with the anti-MIXL1-N antibody. As shown in Fig. 1, the antibody specifically recognized four species for MIXL1 in untreated nuclear extracts. However, the CIAP treatment resulted in the disappearance of two species, α and β at the top and greatly intensified the signals of the lower two bands (γ and δ). Interestingly, the addition of the phosphatase inhibitor orthovanadate restored the four species of MIXL1 proteins on the blot, demonstrating that the two slower isoforms of MIXL1 are phosphatase sensitive. The slowest migrating species of MIXL1 proteins (α) was also lost in the mock reaction without addition of CIAP. The loss could be due to endogenous phosphatase activity from nuclear extracts, as phosphatase inhibitors were not used in both the nuclear extraction and the mock reaction. Together, the phosphatase-sensitive properties indicated that MIXL1 protein is phosphorylated at multiple sites.

### MIXL1 is tyrosine phosphorylated

**Figure 2:**
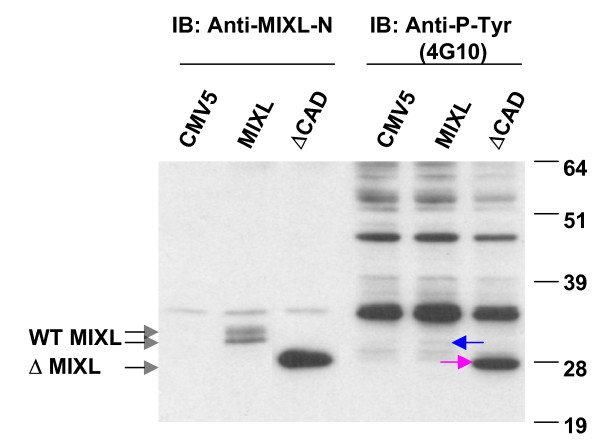
**MIXL1 is phosphorylated on tyrosine residues**. Nuclear proteins were extracted from 293T cells transiently transfected with constructs pCMV5-MIXL1 and pCMV5-ΔCAD. Equal amounts of nuclear extracts (10 μg for CMV5 control and MIXL1 and 5 μg for ΔCAD) were loaded in duplicate into a NuPAGE gel and transferred to PVDF membranes. The blot was divided into two halves and hybridized with either anti-MIXL1-N (1:100) or anti-P-Tyr (mouse monoclonal antibody 4G10 at 1:4000). Anti-MIXL1-N antibody detected two species in transfectants expressing full-length MIXL1 and a strong fuzzy band in transfectants expressing the C-terminal truncation. The second half of the blot probed with 4G10 detected a weak but specific signal (arrow) corresponding to the slower migrating form of full-length MIXL1 and a robust signal (arrow) for the C-terminal truncation.

A phosphorylation prediction program called Netphos 2.0 in the public domain [22] was used to search for potential phosphorylation sites in the MIXL1 protein sequence. By comparing the MIXL1 protein sequence with phosphorylation consensuses for known kinases, the program predicted 14 potential phosphorylation sites including 10 serine residues, 3 threonine residues and 1 tyrosine residue. Since only one tyrosine residue out of two showed potential for phosphorylation, we chose to examine tyrosine phosphorylation first. The phosphotyrosine antibody 4G10, which can specifically detect the tyrosine-phosphorylated proteins, was used to see if MIXL1 is tyrosine phosphorylated. Nuclear extracts prepared from the 293T cells transfected with the constructs pCMV5-MIXL1 (Full-length MIXL1) and pCMV5-ΔCAD (CAD-deleted mutant, ΔCAD) was loaded in duplicate for immunobloting. One half of the blot was hybridized with the antibody 4G10 and the other half with the MIXL1-specific antibody to show protein integrity and the relative position of MIXL1 on the blot. As shown in Fig. 2, the phosphotyrosine antibody 4G10 detected a weak band in the nuclear extracts from the cells with the full-length MIXL1, which was not present in the nuclear extracts with the control vector. The weak species was at a position similar to that of the MIXL1 proteins detected with the anti-MIXL1-N antibody, suggesting that the MIXL1 might be tyrosine phosphorylated in HEK 293T cells. Consistent with this possibility, the phosphotyrosine antibody detected a unique band of much stronger intensity at a position corresponding to mutant ΔCAD, demonstrating that the deletion of the CAD domain might result in increased tyrosine phosphorylation on MIXL1 proteins, possibly due to enhanced stability or easier access to the phosphorylation site(s).

**Figure 3:**
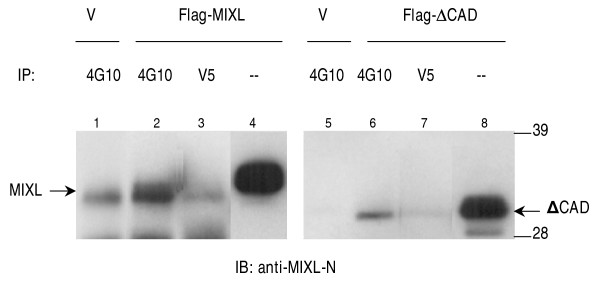
**Immunoprecipitation of phosphorylated MIXL1 by anti-phosphotyrosine antibody 4G10**. Expression constructs pCMV2-flag-MIXL1 and pCMV2-flag-ΔCAD were transfected into HEK 293T cells. 500 μL of nuclear proteins (1μg/μL) from the transfected cells were precleared with 50 μL protein-A-agarose bead slurry (50%v/v) and incubated with 5 μg of the phosphotyrosine-specific antibody 4G10 or the antibody anti-V5 as an isotypic control overnight. The immune complexes were precipitated with 60 μL protein-A-agarose bead slurry (50%v/v). Washed pellets were dissociated and resolved in a NuPAGE gel for immunobloting with the antibody anti-MIXL1-N (1:500). The immunoprecipitation and immunobloting for full-length MIXL1 is shown in the left panel, while mutant ΔCAD in the right panel. A small fraction of both full-length MIXL1 and mutant ΔCAD were immunoprecipitated with the antibody 4G10 (Lane 2 or 6) but not with an isotypic control antibody anti-V5 (Lane 3, or 7). Supernatants from the immunoprecipitation reactions denoted by (-) show the integrity of proteins. A strong band below full-length MIXL1 appeared to be non-specific, as it was also present in the controls. The blot on the left (lanes 1, 2, 3) was exposed to a Kodak film for 30 seconds, while the blot on the right (lanes 4, 5, 6) was exposed for only 5 seconds.

The detection of tyrosine phosphorylation by immunobloting with the phosphotyrosine antibody could not rule out the possibility that the antibody 4G10 was bound non-specifically to the abundant MIXL1 proteins overexpressed in 293T cells. To address this possibility, we examined if MIXL1 proteins could be immunoprecipitated with the antibody 4G10, which specifically binds to phospho-tyrosine residues. The anti-MIXL1-N antibody detected a weak but specific signal for full-length MIXL1 proteins in the immunoprecipitates with 4G10 (Lane 1, 2 and 3 in Fig. 3). Similarly, the mutant ΔCAD was specifically immunoprecipitated with the phosphotyrosine antibody 4G10 (Lane 4, 5 and 6 in Fig. 3). The results demonstrated that the phospho-tyrosine antibody specifically recognized some MIXL1 proteins in nuclear extracts. The immunoprecipitation analysis further confirmed that MIXL1 was tyrosine phosphorylated in 293T cells.

### Amino terminal Tyr20 is phosphorylated

**Figure 4:**
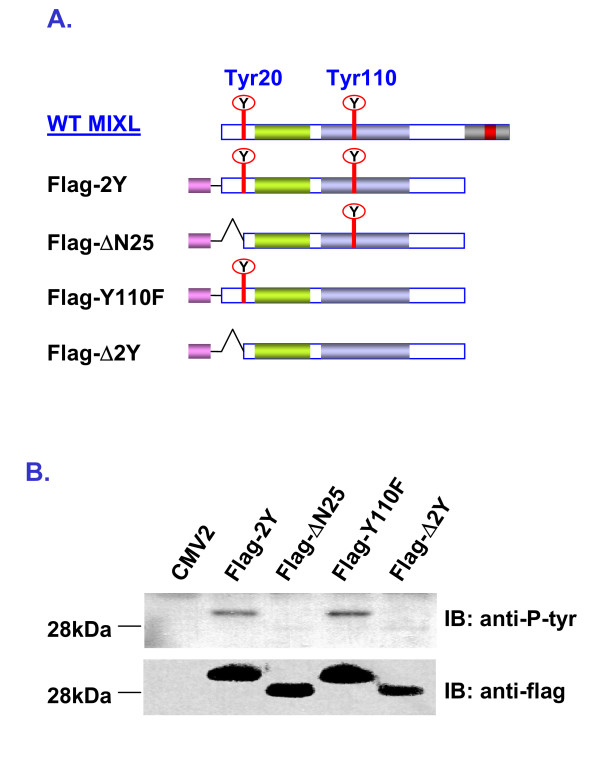
**MIXL1 is phosphorylated on Tyr20**. **A) **Cartoon depicting MIXL1 constructs generated for mutational analysis. Three MIXL1 mutant constructs were derived from the ΔCAD mutant construct pCMV2-flag-ΔCAD (renamed as Flag-2Y in this figure). Each mutant in the constructs has a Flag epitope (a solid gray bar) attached in frame to the amino-terminus and contains either tyr20 or tyr110 or none as illustrated. **B) **Detection of tyrosine phosphorylation. The mutant constructs were transiently transfected into HEK 293T cells. 10 μg of nuclear proteins from the transfectants were resolved on a NuPAGE gel. Tyrosine phosphorylation was examined by immunobloting with monoclonal antibody 4G10 (1:3000). The blot was stripped and re-probed with mouse monoclonal antibody anti-flag (1:300). Tyrosine phosphorylation was detected in both the mutant Flag-2Y and the mutant Flag-Y110F but not in the mutant Flag-ΔN25 as well as the mutant Flag-Δ2Y. The results are consistent with Tyr20 being the target of phosphorylation.

MIXL1 contains only two tyrosine residues. Of these, one is in the amino-terminal domain (Tyr20) and the other is in homeodomain (Tyr110). Therefore, it is easy to determine the tyrosine phosphorylation site(s) in MIXL1 by mutational analysis. Since the mutant ΔCAD showed a much stronger signal for tyrosine phosphorylation than the full-length MIXL1 in western blotting with the antibody 4G10, it was employed in mutation analysis. Based on the ΔCAD mutant sequence, we generated 3 MIXL1 mutants with either a point mutation on the residue Tyr110 or a small amino-terminal deletion including Tyr20 or both (Fig. 4A). Since the antibody anti-MIXL1-N could not detect the amino-terminally deleted mutant proteins, a small epitope Flag was added in frame to the amino-terminal ends of all the three mutants as well as the mutant ΔCAD (Fig. 4A). Nuclear extracts from the 293T cells transfected with the four constructs were prepared for immunobloting with the antibody 4G10. The same blot was stripped and rehybridized with the mouse monoclonal anti-Flag antibody, which detects the Flag epitope. Surprisingly, the antibody 4G10 detected tyrosine phosphorylation on the mutant Flag-Y110F containing the single tyrosine residue Tyr20, similar to that on the mutant Flag-ΔCAD (renamed as Flag-2Y here) with both tyrosine residues (Fig. 4B). In contrast, the antibody 4G10 failed to detect tyrosine phosphorylation on the amino-terminal deletion mutant Flag-ΔN25 containing the single tyrosine residue Tyr110 as well as the mutant Flag-Δ2Y lacking both tyrosine residues, although the protein levels for the four constructs were similar in the nuclear extracts (Fig. 4B). Moreover, the same pattern was detected in the COS-1 cells transfected with those four constructs (data not shown). Thus, these observations suggested tyr20 to be the target of phosphorylation in HEK 293T cells, although this is inconsistent with the prediction by the program Netphos2.0. Whether tyr20 is phosphorylated on endogenous MIXL1 proteins remains to be confirmed.

### Absence of PRD causes a marked reduction in tyrosine phosphorylation

**Figure 5:**
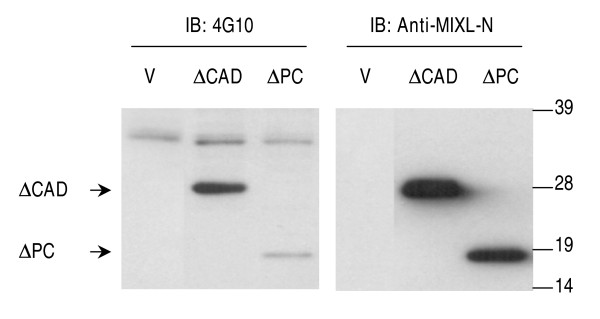
**Tyr20 phosphorylation is diminished in the absence of PRD**. The constructs pCMV5-ΔCAD and pCMV5-ΔPC (lacking both the CAD and PRD) were transfected into HEK 293T cells. 15 μg of the nuclear extracts from the transfectants were resolved on a 10% NuPAGE gel. Tyrosine phosphorylation was detected by immunobloting with monoclonal antibody 4G10. The same blot was stripped and reprobed with anti-MIXL1-N antibody (1:400). Compared to the vector control, the antibody 4G10 detected a weak signal for the ΔPC mutant, although it is much weaker than that for ΔCAD mutant with the intact PRD domain. V-Vector.

Recent studies reveal that the substrate proteins for phosphorylation often contain a docking domain to recruit specific protein kinases [23]. In some instances, the PRD in substrate proteins may compete against the auto-inhibitory interaction between the SH3 domain and catalytic domain, by interacting with SH3 domain in protein kinases such as Src [24]. Since the human MIXL1 contains a PRD immediately downstream of the residue Tyr20 (11 residues downstream from the residue Tyr20), we postulated that the PRD in human MIXL1 might be involved in tyrosine phosphorylation. Thus, we examined the tyrosine phosphorylation on PRD-deleted MIXL1 proteins. Since ΔCAD mutant shows stronger signals for tyrosine phosphorylation, a construct containing the mutant MIXL1 gene with the double deletion of PRD and CAD domains (ΔPC) was generated and transfected into 293T cells. The tyrosine phosphorylation of the mutant ΔPC was determined by immunobloting with the antibody 4G10. As shown in Fig. 5, the tyrosine phosphorylation on the mutant ΔPC was much weaker than that of the mutant ΔCAD, suggesting that the PRD, although not absolutely necessary for Tyr20 phosphorylation, may be important for either achieving full-scale phosphorylation or maintaining the phosphorylation state by preventing dephosphorylation. Future studies with antibodies specific for the Tyr20 phosphorylated protein will elucidate how and when the phosphorylation occurs. More importantly, it will be critical to examine phosphorylation of endogenous MIXL1 in hematopoietic tissues and breast cancer cells. Thus the present report is a first step in elucidating the potential function of the PRD and Tyr20, features unique to avian and mammalian MIXL1.

Since MIXL1 localizes to the predominantly to the nucleus (Guo and Nagarajan unpublished results), the kinase(s) responsible for tyrosine phosphorylation on MIXL1 is likely to be localized in the nucleus. Ten out of the 90 tyrosine kinases encoded in humans, are known to localize to the nucleus (reviewed by Cans et al [25]). Thus the likely candidates are c-ABL1, Wee1, FRK, LYN, FES family (FES and FER) and JAK family (JAK1, JAK2, JAK3, and TYK2). Although the present studies were conducted in HEK293 cells, several of these kinases are expressed in the hematopoietic system. Additionally, since the tyrosine phosphorylation of MIXL1 was not examined in cytoplasm, we could not rule out that MIXL1 is tyrosine phosphorylated in the cytoplasm and translocated into the nucleus.

Unlike serine/threonine phosphorylation, tyrosine phosphorylation on homeodomain proteins is rarely reported to date. Hence the role of tyrosine phosphorylation in the regulation of homeodomain proteins largely remains unclear. The only reported case is the tyrosine phosphorylation of HoxA10 during interferon γ-induced myeloid differentiation. In this case, interferon γ-induced differentiation led to HoxA10 tyrosine phosphorylation in the myelomonocytic cell line U937, which decreased DNA binding of HoxA10 to Pbx-HoxA10 binding sites [26]. However, SHP1 protein-tyrosine phosphatase (SHP1-PTP), which antagonizes myeloid differentiation, decreased tyrosine phosphorylation of HOXA10 homeodomain thereby enhancing HOXA10-mediated repression [27].

A tissue- or cell cycle-specific phosphorylation may alter MIXL1 activity. Future studies will elucidate whether phosphorylation mediated protein-protein interactions due to the unique PRD in human, mouse and chicken Mix-like proteins indeed substitutes for the functional diversity achieved by multiple members in *Xenopus Laevis*.
